# Point-of-care test for cervical cancer in LMICs

**DOI:** 10.18632/oncotarget.7709

**Published:** 2016-02-25

**Authors:** Sulma I. Mohammed, Wen Ren, Lisa Flowers, Bartek Rajwa, Carla J. Chibwesha, Groesbeck P. Parham, Joseph M.K. Irudayaraj

**Affiliations:** ^1^ Department of Comparative Pathobiology, Purdue University Center for Cancer Research, Purdue University, West Lafayette, Indiana 47907, USA; ^2^ Department of Agricultural and Biological Engineering, Purdue University, West Lafayette, Indiana 47907, USA; ^3^ Department of Gynecology and Obstetrics, Emory University, Atlanta, Georgia 30322, USA; ^4^ Bindley Bioscience Center, Purdue University Center for Cancer Research, Purdue University, West Lafayette, Indiana 47907, USA; ^5^ Division of Global Women's Health, Department of Obstetrics and Gynecology, University of North Carolina at Chapel Hill, Chapel Hill, North Carolina 27599, USA

**Keywords:** cervical, cancer, LMIC, Africa, point-of-care

## Abstract

Cervical cancer screening using Papanicolaou's smear test has been highly effective in reducing death from this disease. However, this test is unaffordable in low- and middle-income countries, and its complexity has limited wide-scale uptake. Alternative tests, such as visual inspection with acetic acid or Lugol's iodine and human papillomavirus DNA, are sub-optimal in terms of specificity and sensitivity, thus sensitive and affordable tests with high specificity for on-site reporting are needed. Using proteomics and bioinformatics, we have identified valosin-containing protein (VCP) as differentially expressed between normal specimens and those with cervical intra-epithelial neoplasia grade 2/3 (CIN2/CIN3+) or worse. VCP-specific immunohistochemical staining (validated by a point-of-care technology) provided sensitive (93%) and specific (88%) identification of CIN2/CIN3+ and may serve as a critical biomarker for cervical-cancer screening. Future efforts will focus on further refinements to enhance analytic sensitivity and specificity of our proposed test, as well as on prototype development.

## INTRODUCTION

More than 80% of invasive cervical cancer cases and deaths occur in low- and middle-income countries (LMICs). In Africa, more than 50,000 women die from cervical cancer and approximately 80,400 are diagnosed every year, with incidence variations as high as 5-fold within the continent. In the United States, cervical cancer incidence rate is 7.7 per 100,000 women in 2012; however, prior to the implementation of Papanicolaou (Pap)-based screening, cancer incidence rates (per 100,000 females) in 10 select metropolitan areas in 1947-1948 were of the same order of magnitude (40.1 in whites and 73.1 in nonwhites) as the highest rates found in East Africa today, underscoring the importance of screening and early detection [1, 2].

Organized cervical cancer screening programs in high-income countries use cervical intra-epithelial neoplasia (CIN) as the target lesion. These lesions are now known to result from persistent infection with oncogenic types of human papillomavirus (HPV) [3]. Histopathologically, CIN lesions are subjectively graded into 3 categories depending on the severity of the dysplasia; CIN1 corresponds to mild dysplasia, CIN2 to moderate dysplasia, and CIN3 to severe dysplasia (carcinoma in situ) [4]. Pap cytology tests are the most widely used screening technique, and women with an abnormal Pap test undergo further evaluation using HPV DNA testing and/or colposcopy and biopsy to determine the grade of CIN or to confirm a diagnosis of invasive cervical cancer. The traditional multi-step, laboratory-based screening and diagnosis of cervical disease, dependent for its success on a reliable recall system, makes the Pap test expensive and difficult to sustain in LMICs [5].

Alternative tests to Pap-based cervical cancer screening are visual inspection with acetic acid (VIA) and Lugol's iodine (VILI), and HPV DNA testing. Visual inspection has proved to be valuable as a point-of-care (POC) test that allows for immediate results and treatment. However, it is operator dependent and lacks reliable means of quality assurance. In addition, it has low positive predictive value (precision), resulting in over treatment of a substantial number of women [6, 7]. HPV DNA testing has higher sensitivity but lower specificity than cytology for the detection of CIN2/3 or worse (CIN2/3+). To improve specificity and avoid over-treatment, HPV DNA testing is often combined with Pap cytology to assist in the identification of women who require referral to colposcopy [8]. However, using two screening tests is costly, requires sophisticated laboratory infrastructure, does not provide immediate results in the same way as VIA/VILI screening, and, importantly, may contribute substantially to follow-up losses [9].

Simple, affordable, and accurate screening modalities are urgently needed to improve coverage and effectiveness of cervical cancer prevention efforts globally. Ideally, these tests should be able to be performed at the point-of-care (i.e., rapid) and highly sensitive and specific for identification of CIN2/3+ in order to minimize both false negative and false positive results. In this report, we describe the application of combined approaches using proteomics and lateral-flow immunochromatography (LFIC) technology to (1) identify markers that accurately identify CIN2/3+ and (2) to formulate a simple point-of-care test for cervical cancer screening.

Proteomics is a powerful approach to identifying diagnostic and prognostic biomarkers. It uses a number of protein-analysis techniques that include high-resolution, two-dimensional gel electrophoresis (2-DE), mass spectrometry (MS), and bioinformatics to determine disease-associated biomarkers by observing the expression patterns of proteins, thereby distinguishing between normal and dysregulated physiological processes [10, 11]. Proteomic studies have previously been performed on cervicovaginal lavage fluid, [12, 13] cervical tissue identified as having CIN 2/3 lesions, [14, 15] and cell lines, [16] with many biomarkers identified. However, none of these biomarkers has been sufficiently accurate for cervical cancer screening, and there remains a continuing need for sensitive and specific biomarkers that can be used to improve screening and diagnosis of pre-invasive and invasive cervical cancer, including POC screening algorithms.

LFIC is a simple and widely used technique for the detection of the presence (or absence) of a target analyte in sample mixtures without the need for extensive sample preparation steps [17–25]. A lateral-flow apparatus consisting of a simple membrane with a capture zone and pads through which reagents can be introduced allows the reaction to be completed in a relatively short time (~ 15 minutes) and further enables the in-situ separation of unreacted components via one-step analysis, a key advantage of this format. Additional details of the method are provided in our prior published work [18, 24]. In this work we have used gold-nanoparticle/avidin-biotin constructs to which a number of enzymes can be attached to improve the signal generated. This enhanced LFIC system can detect proteins at very low concentrations.

The goal of this study was to identify a protein biomarker highly sensitive and specific for cervical CIN2/CIN3+, and to develop a simple, colorimetric test for POC cervical cancer screening in LMICs. To this end, we have analyzed the protein-expression profiles of normal, pre-invasive, and invasive cervical cancer tissues using 2-DE followed by protein detection using matrix-assisted laser desorption/ionization time-of-flight mass spectrometry (MALDI-TOF-MS) for identification of differentially expressed proteins as potential biomarkers. The results showed that Valosin-containing protein (VCP) was significantly expressed in CIN2/CIN3 and cervical cancer. VCP was further validated by Western blot and immunohistochemistry. We then developed a test based on the enhanced lateral-flow immunochromatography concept to detect the VCP in cervical tissues.

## RESULTS

### 2D-PAGE and VCP identification

**Figure 1:**
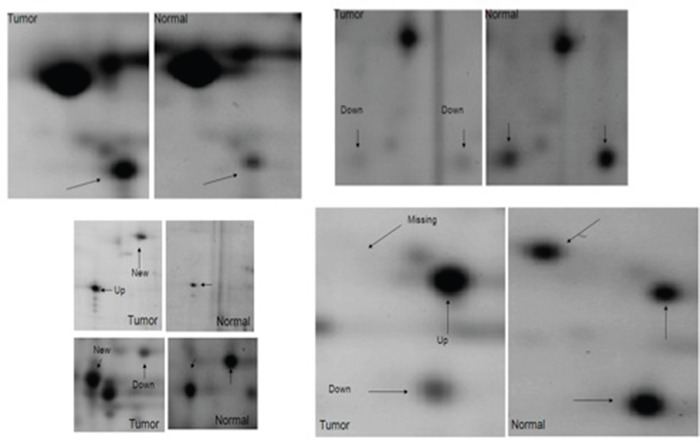
Differentially expressed proteins in cervical carcinoma compared to normal adjacent tissues Selected areas of the spots showing intensity differences between non-neoplastic cervical tissues and neoplastic cervical carcinoma are amplified and indicated by arrows.

In this study, we have characterized and compared protein expression profiles of adjacent non-neoplastic (normal) and CIN2/CIN3, and invasive cervical cancer tissues in order to determine proteins with differentially altered expression that can be identified as potential biomarkers for detection of pre-invasive and invasive cervical cancer. 2-DE and image analysis were used to compare paired adjacent non-neoplastic, pre-invasive, and invasive tumor gels for each individual patient and across patients. A two-fold increase or decrease was arbitrarily defined as up regulated and down regulated, respectively. On average, 1,134 spots were detected per gel. A total of 512 spots across the normal, CIN2/CIN3, and invasive cancer tissues were matched with a match rate of 45%. Approximately 144 spots showed expression levels altered by a factor of at least 2 in comparison to the normal sample as determined by PDQuest. Among these, 85 proteins exhibited increased abundance while 59 showed decreased abundance. Figure 1 shows representative images of some of the spots that were altered in expression, including VCP. Statistical analysis using the Bonferroni correction (p=0.005) was performed to eliminate possible false positives due to multiple, simultaneous comparisons. We determined that the VCP protein was significantly altered in expression between adjacent non-neoplastic, CIN2/CIN3, and invasive cervical tissues (p < 0.005).

### Validation of VCP in cervical tissues using Western blot

**Figure 2:**
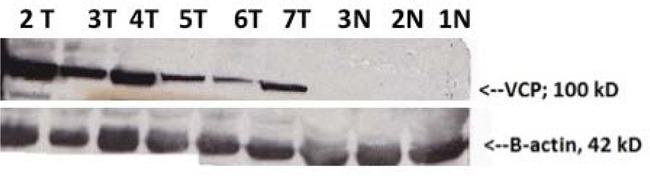
Western Blot validation of VCP proteins detected by proteomics in normal adjacent (N) and cervical precancerous and cancer tissues (T) β-actin loading control is shown in the lower panel (N denotes normal tissues (1N, 2N, 3N), tumor tissues (2T, 3T, 4T, 5T); precancerous tissues (6T and 7T).

Normal, CIN2/CIN3, and invasive cervical cancer tissue from 30 patients (10 in each group) were collected, washed, and homogenized; equal amounts of protein were loaded on SDS-PAGE and expression levels of VCP were determined by Western Blot. Figure 2 shows that VCP is expressed in CIN2/3 compared to invasive cancer. VCP protein was not expressed in the majority of normal tissues.

### Validation of expression of VCP in cervical tissues using immunohistochemistry

**Figure 3:**
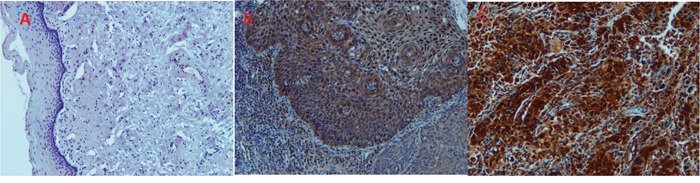
mmunohistochemistry staining of normal **(A)**, CIN3+ **(B)** and cancerous cervical tissues **(C)** with antiserum to VCP protein. No staining is observed for VCP in normal tissues tested (A) while very intense staining is observed in the cytoplasm of CIN3 (B) and tumor cells (C).

To evaluate whether VCP immunoreactivity varies between CIN2/CIN3 and invasive cancer and normal tissues, samples (3-5 slides per patient) from 152 patients were analyzed using immunohistochemical analysis. The immunoreactivity of VCP was classified according to the score methods described in the Materials and Methods section. The immunoreactivity is determined by taking into account the intensity of the signal and the percentage of VCP-stained cells. Representative photographs of VCP staining in invasive cancer, CIN2/3, and normal cervical tissues are shown in Figure 3. Strong staining is shown in the cytoplasm of cancer tissues. Overall expression of VCP was significantly increased in cancer cells compared to normal tissues (Figure 3). Additionally, among the 76 samples classified as “positive” using histopathology (CIN2/3+ samples), 71 were correctly marked as positive employing the VCP-based method. Out of 44 samples scored as “negative,” the VCP approach labeled 5 as positive. The inter-rater agreement between two methods (Cohen's κ) was 0.82, which represents robust agreement. Assuming that the histopathology-based evaluation provided the “ground truth”, the overall accuracy of the VCP-based technique was 0.92 (95% CI: 0.85-0.96). The observed sensitivity was 0.93 and specificity was 0.87. The confusion matrix illustrating the scoring results is presented in Table 1.

**Table 1:** Sensitivity and specificity of VCP in detecting cervical lesions

		Histopathology scoring	
		positive	negative
VCP immunochemistry scoring	positive	71	5
	negative	5	39

### Lateral-flow immunochromatography (LFIC)

**Figure 4:**
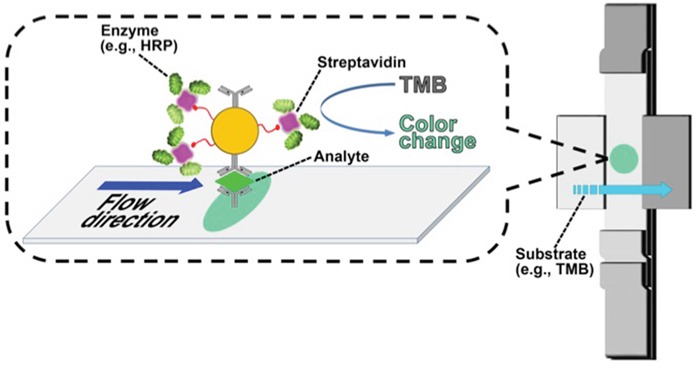
Scheme of enzyme-enhanced LFIC for VCP detection Biotinylated gold nanoparticles tethered with streptavidin-bearing HRP at the capture site in the lateral flow strip to generate a signal upon interaction with TMB.

**Figure 5:**
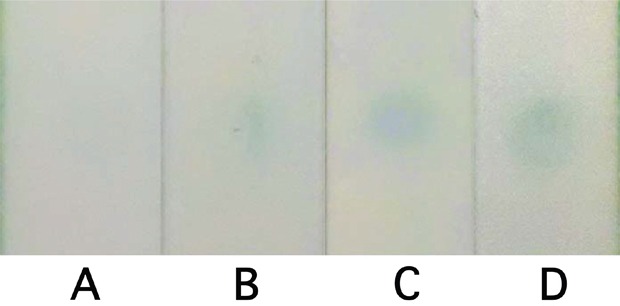
Photographic results of LFIA detection from blank **(A)**, 0.21 ng/ml purified VCP **(B)**, 28.8 ng/ml tissue extractive **(C)**, and 46.3 ng/ml HeLa lysate **(D)**.

We introduce LFIC as a validation and as an onsite screening technique, with the long-term goal of developing a POC assay for cervical cancer screening using this protein (Figure 4). An enzyme-amplified signal was used to detect the selected protein marker, VCP, in three types of samples: purified VCP from cervical cancer tissues, HeLa-cell lysate, and cervical cancer tissue extracts. The photograph of the LFIC strips at the detection step is shown in Figure 5. It can be seen that, compared with the result from a blank, a limit of detection (LOD) as low as 0.21 ng/ml of purified VCP was noted. At the same time, a LOD of 28.8 ng/ml in tissue extract and 46.3 ng/ml in HeLa cell lysate was noted, as shown in Figure 4.

## DISCUSSION

In this work, we have explored opportunities with which to improve cervical cancer screening through identification of novel biomarkers and development of a model for a simple, POC test for cervical cancer screening. To that end, we applied high-throughput proteomic technology and bioinformatics in order to assess the protein profile differences between normal, CIN2/CIN3, and invasive cervical cancerous tissues in order to identify proteins that could serve as biomarkers that would be sensitive and specific for cervical disease. In addition, based on our discovered marker we developed a user-friendly POC technology, LFIC, which would not be reliant on sophisticated laboratory infrastructure or highly trained laboratory personnel.

We have used two-dimensional gel electrophoresis to separate the proteins from normal, CIN2/CIN3, and invasive cancerous cervical tissues. Protein spots on the 2-D gels and image analysis showed different expression patterns between the different groups of tissues studied, indicating protein expressions specific to each set of tissues. Statistical comparison of the protein expressed between groups identified proteins that were significantly decreased, increased, or absent/present in some tissues. Using MALDI/MS identification of these statistically significantly expressed proteins between the groups revealed the following proteins: calreticulin, phophodiesterase PDES 16, tumor rejection antigen, aldehyde dehydrogenase 1A1, RAsAL1 protein, HSP90, phosphoglucomutase 1, vimetin, GRP 78, beta-actin, proapo-A-1 protein, DnaK-type molecular chaperone HSPA1l, mutant beta-actin, apolipoprotein a-1 precursor, albumin, NudE nuclear distribution gene E-like homolog 1, cyclin-dependent kinase inhibitor 1B, keratin, and Valosin-containing protein (VCP, also known as ubiquitin-dependent molecular chaperone p97).

In this report, we have focused on VCP as a potential marker for early detection of cervical cancer. We have shown that VCP is significantly overexpressed in CIN2/CIN3 and in invasive cancer specimens (p < 0.005). In addition, we have confirmed VCP expression in an independent sample of CIN2/3+ tissues by Western blot and immunohistochemistry. Other studies have also showed that VCP is associated with HPV transformation. HPV progression from benign to transforming infection is characterized by an increase of HPV E6/E7 mRNA and protein expression, [26] and VCP has been shown to be regulated by the HPV E6 gene through the E6-E6AP-PTPN3 network [27]. Furthermore, a high correlation between elevated expression of VCP and progression, prognosis, and metastatic potential in pancreatic, hepatic, esophageal, prostate, and colorectal cancers has previously been reported [28–33].

VCP is a member of the AAA class of ATPases found throughout the body and has a wide variety of functions within cells. It is involved in cycle regulation, nuclear envelope formation, Golgi biogenesis, DNA repair, gene expression, cell growth, and the ubiquitin proteasome system [34]. VCP mediates endoplasmic reticulum (ER)-associated degradation, a process by which misfolded proteins localized in the ER lumen or membrane are eliminated through its interaction with several accessory proteins and cofactors. Upon the ubiquitination of misfolded proteins, VCP is thought to extract the targeted proteins from the ER in an ATP-dependent manner maintain their misfolded state until they can be degraded by the cellular proteasome machinery [35] or the aggresome-autophagy pathway [36].

Although distributed in normal cells and needed for many physiological cell processes, p97/VCP ubiquitin is overexpressed in malignant cells in many different types of cancer [28–31, 33]. This could be explained by the fact that VCP is up-regulated by damage-induced protein stress signals that are elevated in cancer cells and that it helps in the clearance of abundant, misfolded/aggregate-prone, and potentially toxic proteins from malignant cells and facilitates their survival [37]. Lack of p97/VCP activity results in accumulation of misfolded, polyubiquitinated proteins leading to apoptosis, as occurs in non-malignant cells.

In order to address some of the limitations in clinical performance and scalability of traditional Pap and/or HPV DNA—based cervical cancer screening, several biomarkers are now commercially available. These include Pre-Tec Proofer (Proofer; Norchip AS, Norway) [38]; APTIMA HPV for detection of E6/E7 mRNA (Hologic, San Diego, CA) [39]; CINtec cytology for detection of P16^ink4a^ protein (Roche Laboratories, Indianapolis, IN) [40]; and ProEX C for detection of topoisomerase IIA and minichromosome maintenance protein 2 (Becton Dickinson, Franklin Lakes, NJ) [41]. However, these biomarkers all suffer heterogeneity in the interpretation of results and non-uniformity in determination of end points.

In our initial laboratory experiments, we have found that VCP is highly sensitive (93%) and specific (89%) for CIN2/3+. We have also demonstrated the overall accuracy to be between 85% and 96% (α= 0.05). Based on these results, we have developed an assay prototype based on LFIC technology. The LFIC assay is easy to use and interpret, with a turn-around time of ~ 15 minutes. As our approach utilizes gold nanoparticles conjugated with antibody and horseradish peroxidase as probes, signals are observable by the naked eye. The simplicity of this assay would allow it to be incorporated into POC screening strategies, including in LMICs, as we anticipate that healthcare personnel with disparate backgrounds and training would be able to use the test safely and effectively. However, additional work is needed to optimize the assay for alternative sample types (e.g., cervical swabs, cervicovaginal lavage specimens, and PreservCyt specimens) and larger studies are needed for robust clinical validation.

In conclusion, our data demonstrate that VCP-specific immunohistochemical staining provides a sensitive and specific identification of CIN2/3+ in cervical tissue specimens and that VCP may represent a useful marker for screening and early detection of pre-invasive and invasive cervical disease. We have also demonstrated proof-of-concept for a POC molecular assay for cervical screening based on LFIC. Future efforts will focus on further improvements in the technology with respect to analytic sensitivity and specificity across sample types, as well as prototype development for larger clinical performance evaluations.

## MATERIALS AND METHODS

### Chemicals and reagents

Urea, trypsin, coomassie brilliant blue r-250, dithiothreitol (DTT), glycerol, glacial acetic acid, alpha-cyano-4-hydroxycinnamic acid (CHCA), acetonitrile (AcN), sodium citrate, sodium carbonate, HAuCl_4_, casein (sodium salt type), Ponceau S, and tetramethyl benzidine (TMB) were purchased from Sigma (St Louis, MO). ReadyStrip was purchased from Bio-Rad (IPG strip pH 4-7; Bio-Rad). VCP antibody was obtained from Abcam (Cambridge, MA). Monoclonal p97/VCP antibody (H0007415-M15 and H0007415-M18) was purchased from Abnova (Taipei, Taiwan). Sulfo-NHS-LC-biotin and streptavidin poly-HRP were purchased from Pierce (Rockford, IL). Biotinylated goat anti-mouse secondary antibody (GAM IgG) conjugated to HRP was purchased from Dakocytomation (Carpinteria, CA). Mayer hematoxylin was purchased from Richard-Allan Scientific (Kalamazoo, MI). Immunohistochemistry reagents were all purchased from Biocare Medical (Concord, CA).

### Human tissue samples and tissue microarray

Fresh-frozen tissues (n=30) and formalin-fixed paraffin-embedded (FFPE) cervical tissues (n=152) were obtained from women who underwent hysterectomy for invasive cervical cancer or benign conditions at Grady Hospital, Atlanta, GA, and Indiana University School of Medicine, Indianapolis, IN. Sample collection and handling were performed under the approval of the Emory University, Indiana University, and Purdue University Institutional Review Boards. Participation was offered to all patients undergoing hysterectomy, and consent was obtained. Prior to removal of tissues for the study, all patients were given a Pap smear test, and if necessary, underwent colposcopy and cervical biopsies in accordance with standard clinical recommendations. Additional FFPE tissues (n=5 cases; 5 slides each) were obtained from Indiana University School of Medicine. Tissue microarray slides each containing 24 tissue sections were purchased from US Biomax Inc (Rockville, MD), 16 of which were normal cervical tissues. Of the 30 fresh-frozen tissues, 10 cervical samples were obtained from patients with invasive squamous-cell carcinoma, 10 were non-neoplastic adjacent tissues, and 10 were from patients with pre-invasive lesions (i.e., CIN2/CIN3). The 120 FFPE tissues included 34 malignant samples, 35 normal tissues, 13 from CIN1 lesions, 10 from CIN2, and 28 from CIN3.

### Protein extraction

Cervical tissues weighing approximately 500 mg were quickly thawed and washed three times in ice-cold “salt-free” phosphate buffer (8 mM Na_2_HPO_4_, 2 mM KH_2_PO_4_) to remove any residual blood. Samples were cut into small pieces and chilled in 10 mL lysis buffer (salt-free phosphate buffer pH 7.5, 0.5% Triton X-100, 1 mM PMSF, 15 mg/mL aprotinin, 10 mg/mL leupeptin, 10 mg/mL pepstatin, 100 mg/mL DNase 1, 25 mg/mL RNase A, 5 mM MgCl_2_). Tissues were homogenized on ice using an electrical tissue homogenizer, and then centrifuged at 20 *g* for 15 minutes at 37° C. The supernatants were transferred to new tubes and protein concentration was determined using an Amersham 2D Quant kit (GE Healthcare Bio-Sciences, Piscataway, NJ). Protein from the above tissue lysates (2.5 mg) was transferred into glass tubes, and precipitated using TCA/acetone precipitation. About 2% carrier ampholyte (pH 8–10.5) was added to the pellet solution (0.4 mL solution containing 9 M urea, 4% Igepal, and 1% DTT) and mixed thoroughly.

### 2-DE and image analysis

200 mg of protein was used to perform 2-DE according to the technique described by Li et al, [42]. The protein samples from adjacent non-neoplastic (n=10), CIN2/CIN3 (n=10), and invasive carcinoma (n=10) were analyzed in triplicate, in addition to pooled protein samples for all carcinomas, and pooled protein samples for all adjacent non-neoplastic tissues. Pooled samples were mixed proportionally so as not to compromise low-abundant proteins. Two hundred micrograms protein from each sample (individual or pooled samples) was placed on first-dimension IEF tube gels containing 3.3% acrylamide, 9 M urea, 2% Igepal CA-630, and 2% ampholyte (BDH pH 4–8). The gels were produced in the laboratory using the LSB Angelique™ Gradient Gel Casting System. The IEF gels were then run simultaneously using progressively increasing voltage (500V for 1 hour, 750V for 1 hour, 1000V for 1 hour, and 1400 V for 18.75 hour) for a total of 28 500 Vh at room temperature. Subsequently, the focused gels were loaded directly onto 2-D slab gels with a linear gradient of 11–19% without equilibration. The slab gels were then run in parallel in a Protean Plus Dodeca Cell (Bio-Rad, Hercules, California) at 87°C for 18 h at 160 V. The slab gels were fixed in 1.5 L of fixing solution (50% ethanol and 2% phosphoric acid) overnight and covered (6 gels/covered box). Gels were then washed and subsequently transferred to 1.5 L of staining solution (30% methanol, 17% ammonium sulfate, and 3% phosphoric acid) for 1 h, followed by the addition of 1g powdered CBB G-250 stain for 96 h. The gels were washed with water and scanned at 95.3-micron resolution using a Bio-Rad GS-800 Calibrated Imaging Densitometer. The resulting images were analyzed using PDQuest software (Bio-Rad, V7.1) on a PC with Windows 2000. The background was subtracted and the peaks for the protein spots were located and counted. Numerous proteins that were uniformly expressed in all gels were used as landmarks to facilitate rapid gel matching. Gels were normalized using PDQuest software. Statistical comparisons between individual protein abundances were conducted by calculation of Student's t-test using the built-in function within PDQuest. In addition, the raw data for each protein spot were exported to Excel (Microsoft, Redmond, WA) for further statistical analysis (see statistical analysis section below).

### Peptide mass fingerprinting

Unique protein spots between each of the sample groups were cut from the gel robotically using 2D Protein Spot Cutter (Bio-Rad) and placed in a 96-well plate. Pieces of blank gels whose mass lists were used as background reference were processed along with other samples. The excised protein spots were destained with 50 mM ammonium bicarbonate, 50% ACN, and 100% ACN, respectively. Samples were reduced with 10 mM DTT, alkylated with 55 mM iodoacetamide, and washed first with 50 mM ammonium bicarbonate and then with 100% ACN. Promega sequence-grade trypsin (10 mL) (12.5 ng/mL in 50 mM ammonium bicarbonate) was added to each sample and incubated overnight at 37° C. The resulting peptides were extracted with 60% ACN and 0.1% TFA, 5% formic acid, and 80% ACN and 0.1% TFA. The peptide extract (1 mL) was mixed with 1 mL of matrix (10 mg/mL CHCA and 0.05% TFA), placed onto each corresponding position on the MALDI target plate, air-dried, and then washed with 10 mL 0.1% TFA for 10 seconds to desalt. The samples were analyzed by MALDI-TOF-MS using M@LDI System (Micromass, UK). Prior to data collection, the instrument was calibrated using peptide standards. Mass lists of all the determined samples were generated by Protein Lynx (Micromass) and filtered against the blank control mass list to eliminate background peaks, using in-house software. The filtered mass lists were submitted to Profound Peptide Mass Database for further analysis. The parameters were as follows: no more than one tryptic miscleavage allowed, cysteine searched as carboxamidomethyl cysteine (157.0215 Da), 50 ppm peptide tolerance and 0.2 Da mass tolerances. A Z-score of 1.65, was obtained. This score corresponds to the 95th percentile and is used as a threshold for a positive identification.

### Western blot analysis

Protein immunoblotting was used to verify and validate the proteomic biomarker candidates. Frozen tissue samples were immersed in lysis buffer containing 1 M Tris (pH 7.6), 5 M sodium chloride (NaCl), 10% sodium azide (NaN_3_), 200 mM EDTA, and 10% Triton X-100. Tissues were homogenized and sonicated on ice and then centrifuged at 10,000 *g* for 10 minutes. The supernatants were transferred to new tubes and protein concentrations were determined with the bicinchoninic acid (BCA) protein assay (Thermo Fisher Scientific, Rockford, IL). Equal amounts of protein (1μg per lane) were electrophoresed by 10% sodium dodecyl sulfate polyacrylamide gel electrophoresis (SDS-PAGE) under reducing conditions and then transferred onto a pure nitrocellulose blotting membrane (Bio-Rad Laboratories, Hercules, CA). The transfer of protein and equal loading in all lanes was verified using reversible staining with Ponceau S (Sigma Chemical, St. Louis, MO) on the NC and β-actin. The membranes were blocked using 5% dry non-fat milk in TBST solution for 30 minutes. Blots were incubated with Valosin-containing protein (VCP) antiserum (Abcam) at a dilution of 1:4000 for 1 h at room temperature. This was followed by the application of biotinylated goat anti-mouse secondary antibody (GAM IgG) conjugated to HRP (Dakocytomation, Carpinteria, CA). The presence of VCP was detected by chemoluminescence, utilizing a gel-documentation system? Antibody specificity was determined by incubating parallel membranes with and without VCP antibody.

### Immunohistochemistry

Formalin-fixed paraffin-embedded sections from the cervical biopsies were processed for VCP immunohistochemistry according to the Biocare protocol using a MACH-4 detection system. Briefly, slides containing tissue sections were deparaffinized in xylene and hydrated in graded concentrations of ethanol. Endogenous peroxidase activity was quenched for 10 minutes (using 0.3% H_2_O_2_) and epitope retrieval was performed by placing the slides in Diva antigen-retrieval solution (Biocare, Concord, CA) for 30 minutes at 95° C in a water bath. Sections were then incubated in primary antibody, monoclonal VCP (1:1, 000 dilution; Abcam, Cambridge, MA), for 30 minutes, followed by 15 minutes in mouse probe and 10 minutes in polymer, and visualized by incubation for 5 minutes in 3,3′ diaminobenzidine (DAB). The sections were then washed and immersed in Mayer hematoxylin (Richard-Allan Scientific, Kalamazoo, MI) for 5 minutes. Finally, the slides were washed and gradually dehydrated and cover slipped after addition of one drop of permanent mounting medium.

We have assessed VCP immunoreactivity semi-quantitatively based on the staining intensity and percentage of stained cells. Expression of VCP was scored as 0 (<5% cells positive); 1+ (6–25% cells positive); 2+ (26–50% cells positive), and 3+ (<50% cells positive). HeLa cells were used as positive controls. VCP staining intensity was scored as “0” (no staining), “1” (low staining), “2” (medium staining), and “3” (high staining).

### Lateral-flow immunochromatography assay

The gold nanoparticles (GNPs) used for the probes were synthesized based on the method reported by Frens et al. [43]. GNP probes for LFIC were prepared based on previously published reports [17, 24]. Briefly, 1 ml GNPs was added to 1 μl of 0.5 M sodium carbonate, 100 μl of 10 mM phosphate buffer (pH 7.4), and 10 μg monoclonal p97/VCP antibody (H0007415-M18), and the obtained solution was kept under gentle stirring for 2 hours. Then 122 μl of 5% (w/v) casein (10 mM phosphate buffer solution, pH 7.4) was injected and the obtained solution was gently stirred at room temperature overnight for blocking. Centrifugation at 10,000 rpm for 10 minutes was performed to remove unbound molecules; the precipitate was washed and redispersed in 1 ml of 10 mM phosphate buffer solution, followed by mixing with 10 μg of sulfo-NHS-LC-biotin. After shaking for 1 hour, 100 μl of 5% (w/v) casein in 10 mM phosphate buffer was added and the obtained solution was gently stirred for 1 hour. The obtained product was centrifuged, washed with 10 mM PBS, and redispersed in 100 μl of 10 mM PBS buffer with 5% (w/v) casein.

The strips for Lateral Flow immunochromatography (LFIC) detection were prepared with 0.2 μg monoclonal p97/VCP antibody (H0007415-M15) fixed on the nitrocellulose membrane and dried at 37° C. Detection of the target was performed following the reported approach [24]. During LFIC detection, the sample absorbent pad of the strip was loaded with 100 μl sample mixed with 0.2 μl GNP probe and 0.2 μl streptavidin poly-HRP to initiate the immunoreaction between the GNP probe-labeled VCP and the VCP antibody (p97/VCP antibody, H0007415-M15) fixed on LFIC strip. After 15 minutes, the strips were fixed with 2 more absorbent pads in the cross-flow direction and 60 μl water was added to wash off the unbound reactants on the strip during cross-flow. 30 μl TMB was added to one of the additive pads for signal generation and then 60 μl water was used to stop the reaction. The color signals observed on the LFIC strips were utilized to quantify the VCP in the samples based on the developed calibration plot.

### Statistical analysis

For a selected spot in the gels, a two-sample paired t-test was used to compare invasive carcinomas and their paired non-neoplastic tissues or pre-invasive lesions. The analyzed variable was the log of the spot intensity. If the spot was not present, ‘0’ was used to signify the lack of intensity. Bonferroni correction was used to adjust for multiple comparisons, where the cut-off value was p = 0.005. Bonferroni correction is a multiple-comparison correction used when several dependent or independent statistical tests are being performed simultaneously. Spots that were noted to be significantly different between the three issues types were selected for further consideration as potential biomarkers.

The observed VCP immunoreactivity rated as described above was compared to the results of established histopathology-based diagnosis. For the purpose of this comparison a binary classification scheme was created: VCP responses rated 2+ and 3+ were categorized as “positive,” whereas those rated 0 and 1+ were considered negative. The histopathological samples scored as CIN2 or CIN3 or cancer were considered “positive,” and samples designated as “normal” or CIN1 were considered “negative.” The results were represented using a confusion matrix (or contingency table), and we calculated overall accuracy with a 95% confidence interval, Cohen κ, sensitivity, and specificity of VCP for CIN2/3+.
